# Convexity Bias and Perspective Cues in the Reverse-Perspective Illusion

**DOI:** 10.1177/2041669516631698

**Published:** 2016-02-29

**Authors:** Joshua J. Dobias, Thomas V. Papathomas, Vanja M. Vlajnic

**Affiliations:** Department of Psychology and Counseling, Marywood University, Scranton, PA, USA; Department of Biomedical Engineering and Laboratory of Vision Research, Rutgers University, Piscataway, NJ, USA; Department of Statistics, The Pennsylvania State University, University Park, PA, USA

**Keywords:** Three-dimensional shape, reverse perspective, convexity bias, linear perspective, fixation location, visual context

## Abstract

The present experiment was designed to examine the roles of painted linear perspective cues, and the convexity bias that are known to influence human observers’ perception of three-dimensional (3D) objects and scenes. Reverse-perspective stimuli were used to elicit a depth-inversion illusion, in which far points on the stimulus appear to be closer than near points and vice versa, with a 2 (Type of stimulus) × 2 (Fixation mark position) design. To study perspective, two types of stimuli were used: a version with painted linear perspective cues and a version with blank (unpainted) surfaces. To examine the role of convexity, two locations were used for the fixation mark: either in a locally convex or a locally concave part of each stimulus (painted and unpainted versions). Results indicated that the reverse-perspective illusion was stronger when the stimulus contained strong perspective cues and when observers fixated a locally concave region within the scene.

## Introduction

When viewing reverse-perspective stimuli (Wade & Hughes, 1999), painted linear perspective cues can compete with bottom-up monocular (motion parallax, shading, lens accommodation) and binocular (disparity, vergence angle) depth cues, thus creating a bistable percept: The bottom-up cues favor the veridical depth arrangement, whereas the perspective cues elicit a depth-inversion illusion (DII), in which distant points on the stimulus appear to be closer than near points and vice versa (Cook, Hayashi, Amemiya, & Suzuki, Leumann, 2002; Cook, Yutsudo, Fujimoto, & Murata, 2008; Dobias & Papathomas, 2013, 2014; Hayashi, Umeda, & Cook, 2007; Papathomas, 2002, 2007; Papathomas & Bono, 2004; Rogers & Gyani, 2010; Sherman, Papathomas, Jain, & Keane, 2011; Wagner, Ehrenstein, & Papathomas, 2008). Similar cases of DII can occur with either a hollow mask (Gregory, 1970, 1997; Hill & Bruce, 1993, 1994; Hill & Johnston, 2007; Matthews, Hill, & Palmisano, 2011; Papathomas & Bono, 2004) or merely a hollow oval shape (“hollow-potato”; Hill & Bruce, 1994; Johnston, Hill, & Carman, 1992). The causes of DII are not fully understood, but evidence suggests that linear perspective and texture gradients (Dobias & Papathomas, 2014; Papathomas, 2002; Rogers & Gyani, 2010; Wade & Hughes, 1999), face-specific familiarity (Gregory, 1970, 1997; Hill & Bruce, 1993; Hill & Johnston, 2007), and the bias for convexity (Hill & Bruce, 1994; Langer & Bulthoff, 2001; Ramachandran, 1995; Sherman et al., 2011) play a role in DII.

In this brief report, we examine the roles of linear perspective and convexity in the reverse-perspective illusion using a 2 (Type of stimulus) × 2 (Fixation mark position) design. First, to study the role of perspective, we use two types of reverse-perspective stimuli: painted and unpainted; the difference in the strength of the DII between the two conditions will provide evidence for the role of painted perspective cues. Second, to examine the role of convexity, we ask observers to fixate two different positions by placing the fixation point either in a locally convex or a locally concave part of the same stimulus (painted or unpainted). If a convexity preference exists, fixating on a locally concave part would increase the strength of the DII as compared with fixating on a locally convex part of the stimulus. In the former case, a convexity bias would tend to invert the depth of the concave part, thus encouraging the DII, whereas in the latter case, a convexity bias would tend to obtain a veridical convex surface, thus reducing the DII strength.

## Results

**Figure 1.**
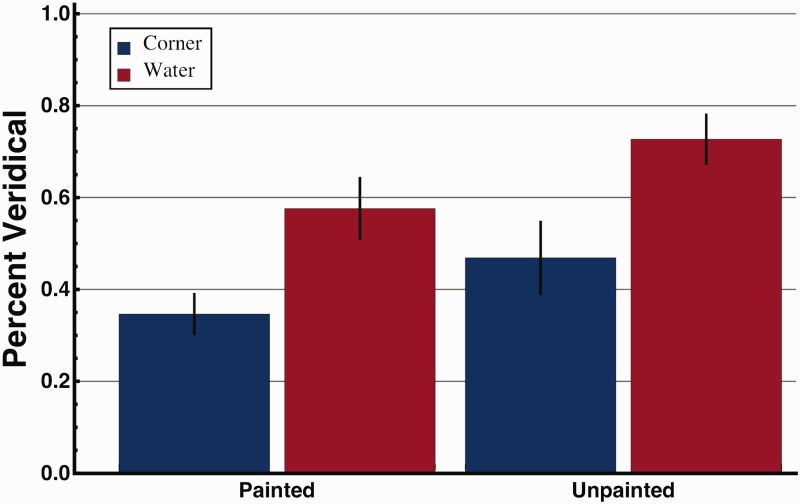
Average proportion of time that observers spent perceiving the veridical shape of the reverspective stimulus when fixated at the corner of the center building (blue) versus the top of the truncated pyramid (red) for painted and unpainted stimuli. Error bars represent ±1 *SEM*.

The average predominance or strength of the veridical percept for each of the four stimulus conditions is shown in Figure 1. Similarly, the average strength of the illusory percept can be calculated by taking one minus the strength of the veridical percept. A 2 Stimulus (painted vs. unpainted) × 2 Fixation position (central concave corner vs. convex “water” region) repeated-measures analysis of variance (ANOVA) was conducted to determine differences between conditions. Finally, planned *t* tests were conducted to determine differences between the average predominance values for the central corner versus water fixation position for each stimulus. The ANOVA showed a main effect of stimulus (*F* = 5.268, *p* = .038, η^2 ^= 0.273), wherein observers perceived the veridical shape more when viewing the unpainted stimulus. Further, results showed a main effect of fixation position (F = 9.339, p = 0.009, *η*^2 ^= 0.40) where observers perceived the veridical shape more when fixated at the top of the convex truncated pyramid (water location). There was no stimulus-by-fixation position interaction (*F* = 0.144, *p* = .710, η^2 ^= 0.010). When comparing fixation positions, planned *t* tests showed that the illusion was weaker (predominance of veridical perception was higher) when fixating at the water (the locally convex truncated pyramid part of the stimulus) than at the central corner (the locally concave part) for both painted (*t*_14_ = 2.84, *p* = .026) and unpainted (*t*_14_ = 3.08, *p* = .008) stimuli. Despite not containing a painted water scene, we will continue to describe the top of the truncated pyramid for the unpainted stimulus as a “water” location in the same way as it is labeled for the painted stimulus.

## Discussion

Results support the predicted preference for convexity. Further, the strength of the illusion was weaker when linear perspective and texture cues were reduced for the plain white reverse-perspective stimulus. When fixating the “water” mark at the top of the truncated pyramid, which is at 256 (268−12) cm, the distance from the observer is about 4.5% shorter than when fixating the central building corner (at 268 cm). The strength of reverse-perspective illusions has been shown to decrease as the viewing distance decreases (Dobias & Papathomas, 2013; Papathomas, 2002; Rogers & Gyani, 2010). It is unlikely, however, that this small 4.5% decrease in viewing distance was responsible for the significant decrease in illusion strength, which was 35.4% (0.42–0.65)/0.65] for the painted and 49.1% (0.27–0.53)/0.53] for the unpainted stimuli in this experiment. For comparison, Dobias and Papathomas (2013) reported that decreasing the viewing distance by a factor of 50% (from 535 to 267.5 cm) for the same painted stimulus used in the present experiment, decreased the illusion strength by only 14.7%. Thus, the difference in illusion strength is likely due, to a major extent, to the convexity bias.

## Method

**Figure 2.**
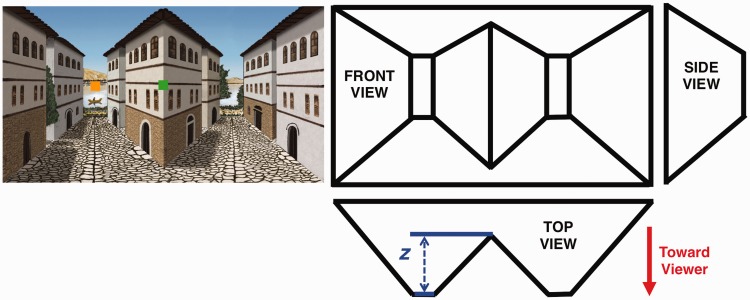
Left: Front view of the painted stimulus. Right: Front, top, and side views of the reverspective stimuli. Fixation locations are marked with a square, in the same way as they were marked in the experiment, either in the center of the stimulus (corner of central building, green) or at the top of the truncated pyramid (water, orange). Note: The scale of fixation marks was slightly smaller in the actual experiment.

A total of 15 naïve observers (ages 18–24) were recruited at Rutgers University and received monetary compensation for their time. Each observer reported normal or corrected-to-normal visual acuity and had normal stereopsis as determined by tests with random-dot stereograms (Julesz, 1971, 2006). Written consent was obtained from each observer and experimental procedures were conducted in compliance with the standards set by the IRB at Rutgers University. Experimental stimuli consisted of the reverspective stimulus “Kastoria” (Figure 2) that has been described previously (Dobias & Papathomas, 2013, 2014; Wagner et al., 2008). A purely geometrical 3D representation of both painted and unpainted stimuli is shown in the right part of Figure 2 in the form of front, top, and side views. The left part of Figure 2 shows the front view of the painted stimulus that contains rich pictorial perspective cues. The unpainted stimulus appeared as in the right part of Figure 2 with the exception that the edges defining the boundaries of each plane were not painted black. As described earlier, both reverspective stimuli could be perceived either in the true (veridical) or the depth-inverted (illusory) state. For the veridical percept, the two truncated pyramids in the stimulus were correctly perceived to protrude toward the viewer causing the center building to appear concave. For the illusory percept, however, the stimulus appeared to be a scene in which two streets recede into the distance on each side of the central convex building. Both stimuli had a height of 42.5 cm, a width of 71.3 cm, and a depth (*z* in Figure 2) of 12 cm.

Observers sat facing each stimulus at a distance of 268 cm, measured from the corner of the central building, with their chin placed on a chin rest to maintain head position. Viewing distance was selected based on previous work (Dobias & Papathomas, 2013), in which observers exhibited a bistable percept with roughly 58% dominance of the illusory percept when viewing the same painted stimulus. While the exact location of eye fixation was not monitored, observers were asked to keep their eyes focused on the fixation mark and to move their eyes as little as possible. The fixation locations were either at the concave corner of the center building (green square, Figure 2) or at the top of the convex truncated pyramid (orange square). Observers remained within the concave (or convex) part of the stimulus even if they temporarily moved their gaze as much as 2.85° away from the green (or orange) fixation mark; this large margin, combined with their report of maintaining fixation, obviated the need to monitor eye movements. The fact that eye movements were not recorded did also eliminate the ability to monitor vergence angle. Vergence has been shown to change as the perceived fixation location changes within the bistable physical reverspective stimulus (Wagner et al., 2008). However, as described above, these small changes in perceived fixation distance and vergence angle do not explain the large changes in illusion strength. Further, despite the finding that changes in vergence after a saccade occur based on disparity cues in spite of the perceived slant of a bistable surface (Wismeijer, van Ee, & Erkelens, 2008), observers in our task were asked to fixate throughout each trial and, therefore, likely achieved stable vergence angles in accordance with the perceived distance of the fixation location, as predicted by Wagner et al. (2008). Stimuli were affixed to a wall and were lit from all sides to avoid shadows. Each experimental session consisted of eight 3-minute trials in which the observer viewed each of the four combinations (2 Stimulus types × 2 Fixation mark positions) twice. While fixated on one of the fixation marks (orange or green), observers were instructed to press and hold one of two keys on a keyboard to indicate the perceived shape of the reverspective throughout each 3-minute trial: If the center building appeared to be “popping out” or “caved in,” observers were asked to press and hold the left or right arrow key, respectively. The order of stimulus presentation was randomized for each observer. *Mathematica 8.0.1.0* (Wolfram Research, Inc., 2010) was used to record the duration that the observer pressed each of the two buttons and to compute the predominance of the veridical percept (percentage of the total viewing time in that percept). Predominance is a common measure of the strength of the veridical percept (Dobias & Papathomas, 2013; Papathomas & Bono, 2004; Sherman et al., 2011). Data for each observer are the average of the two predominance values for each of the 3-minute trials for which the observer viewed each of the four conditions. A sound (short beep) indicated the beginning of each 3-minute viewing trial. Once the trial was complete, a second sound indicated the end of the trial.
